# Advanced Practice Providers Versus Physicians in Emergency Department Abdominal Pain: Comparison of Length of Stay and Return Visits

**DOI:** 10.1111/acem.70318

**Published:** 2026-05-08

**Authors:** Emad Awad, Shilpa Raju, Micah Ownbey, Paul Kim, Austin Johnson

**Affiliations:** ^1^ Department of Emergency Medicine University of Utah Salt Lake City Utah USA; ^2^ U‐Emerge University of Utah School of Medicine Salt Lake City Utah USA

**Keywords:** abdominal pain, advanced practice providers, emergency department, health services research, length of stay, nurse practitioners, physician assistants, physicians, return visits

Emergency department (ED) crowding is a major systems challenge [1]. To meet rising volumes, EDs increasingly deploy advanced practice providers (APPs: nurse practitioners and physician assistants) [2, 3]. Although prior research shows APPs provide safe care [2, 3, 4], their impact on efficiency for specific high‐acuity complaints like abdominal pain is less clear, particularly outside fast‐track settings [5, 6]. We compared ED length of stay (LOS) and 7‐day return visits for abdominal pain patients initially managed by APPs versus physicians in an integrated, high‐acuity setting.

We conducted a retrospective observational study of adult ED visits at two EDS We conducted a retrospective observational study of adult ED visits at two EDS within a single academic health system (January 2020–January 2025). Both sites use an integrated staffing model where physicians and APPs work in all areas; all APP charts require attending physician co‐signature. We included patients aged ≥ 18 years with a triage chief complaint of abdominal pain. Flank pain was included when documentation suggested intra‐abdominal etiology. We excluded non‐painful gastrointestinal complaints without abdominal pain as the primary presenting symptom (e.g., isolated vomiting or gastrointestinal bleeding), trauma, pregnancy, and low‐acuity (ESI 4/5) visits, which represented < 0.5% of potential cases. Only the first visit per patient was analyzed. Provider type (APP or physician) was assigned to the first clinician to sign a note or place orders. Care involving residents was attributed to the supervising attending. The primary outcome was ED LOS (triage completion to disposition entry). The secondary outcome was unplanned 7‐day return among discharged patients.

We used descriptive analyses to summarize patient characteristics. For bivariate comparisons between APP‐ and physician‐initial visits, we used the Student's *t*‐test or Wilcoxon rank‐sum test for continuous variables and the chi‐squared or Fisher's exact test for categorical variables.

Multivariable analyses were performed to evaluate the independent association between provider type and outcomes. LOS was modeled using generalized linear models (GLM) with a Gamma distribution and log link, chosen to account for the right‐skewed distribution of time outcomes. Because disposition status strongly influences LOS, we conducted a prespecified subgroup analysis restricted to discharged patients, using the same covariates.

The secondary outcome (7‐day return visits) was modeled using multivariable logistic regression, restricted to discharged patients.

All models included age, sex, race, triage ESI, triage vital signs, ED site, door‐to‐doc time, and whether CT abdomen/pelvis was performed. This adjustment controls for a major time‐intensive element of the workup that could confound efficiency comparisons [7]. As a retrospective study of all eligible visits, an a priori power calculation was not performed; post hoc, the sample provided > 80% power to detect an absolute difference in return rates of ≥ 1.5%.

We identified 17,046 visits: 14,155 (83.0%) physician‐led and 2891 (17.0%) APP‐led. APP patients were younger, less frequently triaged as ESI‐2 (4.6% vs. 10.3%; *p* < 0.001), and far more likely to be discharged (93.0% vs. 50.2%) (Table 1). CT utilization was similar (7.2% APP vs. 7.5% physician, *p* = 0.10). The overall median LOS was 6.1 h.

**TABLE 1:** Cohort characteristics and outcomes for abdominal pain visits by initial provider type.

Characteristic	Physician (*n* = 14,155)	APP (*n* = 2891)	*p*
Demographics			
Age, mean (SD)	48.1 (18.9)	44.3 (17.8)	< 0.001
Female, *n* (%)	8724 (61.6)	1914 (66.2)	< 0.001
White race, *n* (%)	9951 (70.3)	2002 (69.2)	0.27
Acuity and vitals			
ESI 2, *n* (%)	1461 (10.3)	134 (4.6)	< 0.001
ESI 3, *n* (%)	12,694 (89.7)	2757 (95.4)	< 0.001
Triage vital signs			
SBP, mean (SD)	133.8 (21.2)	133.4 (19.9)	0.38
HR, mean (SD)	87.9 (18.4)	85.8 (17.3)	< 0.001
Process and disposition			
Door‐to‐provider time, median (IQR)	21.3 (9.5–62.0)	23.6 (10.6–68.5)	< 0.001
CT abdomen/pelvis, *n* (%)	1061 (7.5)	208 (7.2)	0.10
Main ED location, *n* (%)	10,916 (77.1)	2376 (82.2)	< 0.001
Discharged, *n* (%)	7104 (50.2)	2691 (93.0)	< 0.001
Admitted, *n* (%)	6157 (43.5)	86 (3.0)	< 0.001
Outcomes			
Adjusted LOS difference (min)			
Full cohort	Reference	−24.2 (−29.6 to −18.8)	< 0.001
Discharged only	Reference	+14.1 (7.7–20.6)	< 0.001
7‐day return, adjusted OR (95% CI)	Reference	1.09 (0.87–1.36)	0.46

*Note:* Models for LOS (Gamma GLM) and return visits (logistic regression) were adjusted for age, sex, race, ESI, triage vital signs, door‐to‐provider time, ED location, and CT abdomen/pelvis use. Abbreviations: APP, advanced practice provider; ESI, Emergency Severity Index; SBP, systolic blood pressure; HR, heart rate; LOS, length of stay; OR, odds ratio.

In the adjusted model for the full cohort, APP‐led visits were associated with a significantly shorter LOS. The adjusted mean LOS for APP‐led visits was 6.4% shorter than for physician‐led visits (adjusted mean ratio [aMR] 0.94; 95% CI 0.93–0.95; *p* < 0.001), equivalent to approximately 24 min faster. However, in the prespecified subgroup of discharged patients only, after adjusting for covariates including CT use, APP‐led visits were associated with a 3.9% longer mean LOS (aMR 1.04; 95% CI 1.02–1.06; *p* < 0.001), or about 14 min slower.

Of the 9795 discharged patients, 419 (4.3%) had an unplanned return within 7 days. Return rates did not differ between provider types (4.2% physician vs. 4.4% APP; *p* = 0.65). In adjusted logistic regression, provider type was not associated with 7‐day return (adjusted odds ratio 1.09; 95% CI 0.87–1.36; *p* = 0.46).

Our results reveal nuanced effects of provider type on ED throughput for abdominal pain. The shorter adjusted LOS for APPs in the full cohort is likely explained in part by case‐mixed differences, as physicians‐led visits were far more likely to result in admission. In routine practice, this pattern likely reflects physicians caring for a greater proportion of higher‐acuity or more diagnostically complex patients and those with a higher anticipated need for hospitalization. The finding of slightly longer APP LOS among discharged patients, after accounting for CT use, suggests that when patient acuity and key resource utilization are similar, physician care may be modestly more efficient for patients ultimately sent home. This adjustment for CT use helped account for an important potential confounder in LOS comparisons [7]. Importantly, 7‐day return visit rates were identical, supporting comparable short‐term safety [8, 9].

This study has limitations. Its single‐system design may limit generalizability. Although we adjusted for CT use, residual confounding from other unmeasured factors (e.g., consultant use) is possible. The observational design precludes causal conclusions.

In conclusion, in this high‐acuity abdominal pain cohort, APPs were associated with shorter overall LOS but slightly longer LOS among discharged patients after adjusting for CT use, with no difference in 7‐day return visits. These findings support the complementary role of APPs in ED staffing models and underscore the importance of adjusting for key diagnostic practices when evaluating provider efficiency.

This study has limitations. Its single‐system design may limit generalizability. Although we adjusted for CT use, residual confounding from other unmeasured factors (e.g., consultant use) is possible. The observational design precludes causal conclusions.

In conclusion, in this high‐acuity abdominal pain cohort, APPs were associated with shorter overall LOS but slightly longer LOS among discharged patients after adjusting for CT use, with no difference in 7‐day return visits. These findings support the complementary role of APPs in ED staffing models and underscore the importance of adjusting for key diagnostic practices when evaluating provider efficiency.

## Author Contributions

E.A.: conceptualization, data curation, formal analysis, methodology, writing – original draft. S.R., M.O., P.K.: data curation, methodology, writing – review and editing. A.J.: supervision, writing – review and editing. All authors read and approved the final manuscript.

## Funding

The authors have nothing to report.

## Conflicts of Interest

The authors declare that they have no known competing financial interests or personal relationships that could have appeared to influence the work reported in this paper, with the following exception: A.J. is the founder, advisor to, and a shareholder in Certus Critical Care, and serves on the board of Titin KM Biomedical.

## Data Availability

The data that support the findings of this study are available from the corresponding author upon reasonable request.
